# Uptake and determinants of private health insurance enrollment in a country with heavily subsidised public healthcare: A cross-sectional survey in East Coast Malaysia

**DOI:** 10.1371/journal.pone.0278404

**Published:** 2023-01-17

**Authors:** Mohd Adli Abd Khalim, Surianti Sukeri

**Affiliations:** Department of Community Medicine, School of Medical Sciences, Universiti Sains Malaysia, Kubang Kerian, Kelantan, Malaysia; IGMC: Indira Gandhi Medical College, INDIA

## Abstract

Malaysia’s subsidised public healthcare system is heavily reliant on government funding. Increasing the uptake of private health insurance (PHI) would alleviate the financial burden on public healthcare facilities caused by high patient loads. The study aimed to determine the uptake of PHI and its associated factors among the East Coast Malaysian populations. A cross-sectional online survey was conducted between February and June 2021. Proportionate stratified sampling was applied to select 1138 participants, and logistic regression was performed to determine the factors associated with PHI uptake. The proportion of the study samples that purchased PHI was 54.3%. Enrolment of private health insurance was associated with working in the public sector (aOR: 6.06, 95% CI: 2.65, 13.88) and private sector (aOR: 6.27, 95% CI: 2.65, 14.85), being self-employed (aOR: 9.23, 95% CI: (3.59, 23.70), being in the middle 40% household income percentile (aOR: 2.74, 95% CI: 1.95, 3.85) and top 20% household income percentile (aOR: 4.42, 95% CI: 2.87, 6.80), and living in urban areas (aOR: 1.31, 95% CI: 1.01, 1.70). Even in the presence of subsidised public healthcare, the high proportion of PHI uptake reflects a demand for private health insurance. The study suggests that PHI should be promoted among those who are employed or self-employed, the middle- and high-income groups, and urban residents. The findings may be beneficial for the government and insurance companies to improve strategies to enhance PHI uptake among these population.

## Introduction

Malaysia is an upper-middle-income country that adopts the Beveridge health financing model, which is characterised by the provision of subsidised health services to all citizens through government revenue [1, 2]. Government employees and their dependents, government pensioners, and the disabled continue to receive free public healthcare. Maternal and child health services, as well as treatment for certain infectious diseases, are provided at no cost. Primary care at public health clinics costs only USD0.24, which includes medication [1]. The total health expenditure accounted for 4.3% of the country’s gross domestic product with the top three sources of health financing being the Ministry of Health (44.9%), out-of-pocket expenditures (35.0%), and private health insurance (PHI) (7.6%) [2]. The average life expectancy of Malaysians in 2021 was 75.6 years [3], higher than the estimated global average of 72.7 years [4]. However, the public health system is currently overburdened and underfunded [5] with even the wealthy receiving subsidised care. Heavy reliance on out-of-pocket expenditures expenses may lead to catastrophic health costs and a decline in individual economic status. Given that health reform is currently not an option for Malaysia, it may be necessary to reconsider healthcare financing through a voluntary pre-payment scheme, such as PHI. Increasing the adoption of PHI among those who can afford it may prevent a significant amount of out-of-pocket healthcare spending and, in the long run, avert households from incurring catastrophic costs. Private health insurance has also been shown to improve healthcare financing by shifting demand from the public to the private sector and reserving public resources for underprivileged populations [6].

The proportion of PHI enrollment in 1983 was estimated to be around 1.5% of the population in Malaysia. In 1995, the figure had increased tenfold [7]. Private health insurance is commonly referred to as a "medical card" in Malaysia. The term refers to a card that entitles the policyholder to cashless hospitalisation at any of the insurance provider’s private panel hospitals [8]. All PHI operators in Malaysia are regulated by the Central Bank of Malaysia under the Insurance Act 1996 [9]. Private health insurance is risk-rated, which means that premiums will vary depending on the risks that the insurer is willing to take on. The functional type of PHI in Malaysia is supplementary in nature. It provides faster access to inpatient care in private hospitals, a wider range of health care providers, and improved amenities [10]. The annual limit, lifetime limit, ward categories, coverage of pre-hospitalisation diagnostic tests, post-hospitalisation treatment, surgical fees, emergency accidental outpatient treatment, cancer and dialysis outpatient treatment, and daily cash allowance are standard features on all medical cards. Private health insurance policies are distinguished by the fixed claimable amount for each component. The main disadvantage of a PHI is that it is only applicable to inpatient care, and ward admission will require insurance provider approval [8].

The latest available data reported the proportion of PHI uptake in Malaysia at 20.5% in 2019 [11]. However, the demand for PHI in the less developed region and the determinants of PHI uptake in Malaysia remain unknown. A review of the literature showed that the uptake of PHI was related to age, gender, educational level, marital status, ethnicity, household income, employment status, and strata. Individuals in the age group of 35–44 years old were more likely to acquire PHI, while those exceeding 60 years of age had the lowest proportion of PHI coverage [12, 13]. The association between gender and PHI purchasing is currently inconclusive. Women in Brazil had higher insurance health coverage [12], while in Namibia, the proportion of PHI ownership was higher among men [14]. However, studies in China and India showed that the proportions of PHI ownership among males and females were equivalent [13, 15, 16]. A higher educational level is usually associated with better employment with promising salary prospects and better health literacy that increases the likelihood of obtaining accurate information on health status and services. Numerous studies have shown that education level is related to a higher likelihood of purchasing PHI [13, 16, 17]. Married individuals were more likely to purchase PHI in Korea, Namibia, and China [14, 16, 18] compared to those who were single or widowed. Based on the limited literature on PHI uptake in Malaysia, there was no data on PHI purchases among the three major ethnic groups. However, a study conducted in Penang by Shafie and Hassali (2013) regarding the willingness to pay for voluntary community-based health insurance found that Malaysian Chinese were willing to pay more compared to other ethnicities [19].

A positive relationship between household income and PHI uptake was also observed [12, 14]. Understandably, wealthier households had more disposable personal income to acquire PHI. Stable employment was an important factor in deciding whether to acquire PHI. A higher proportion of PHI ownership was observed among those who were military personnel and those who worked in public and private sectors [12]. Among those who were currently working, the types of enterprises also had a certain influence on the purchase of PHI. For instance, employees of state-owned enterprises and private/foreign-owned enterprises were more likely to acquire PHI compared to other types of enterprises [13]. The proportion of PHI uptake was also significantly higher in the urban area [13, 14].

Despite the above evidence, there is an absence of data on consumer behaviour towards the voluntary purchase of PHI in Malaysia. The lack of data will interfere with the government’s agenda of promoting PHI uptake to reduce the burden on the public healthcare system. For these reasons, this study aims to measure the uptake of PHI and its associated factors among the East Coast Malaysian populations. The findings on the determinants of PHI uptake may be used towards improving the health financing strategy in Malaysia by increasing the uptake of PHI based on the demands and characteristics of the current PHI enrollees. It is high time for such an initiative to be pursued since the COVID-19 pandemic has resulted in a significant burden on public healthcare facilities and an even greater financial strain on the government. Furthermore, the study findings may also provide additional insights for the future implementation of a national health insurance system in Malaysia.

## Materials and methods

### Participants and study design

A cross-sectional online survey was conducted from February to June 2021, involving states in Peninsular Malaysia facing the South China Sea, namely Kelantan, Terengganu, and Pahang. These states were chosen because of the low uptake of PHI compared to other states in Malaysia [11]. The source population were residents in the East Coast region. The inclusion criteria for the study were citizens aged 18 years or above.

Using the single proportion formula based on a national estimate of 20.5% of PHI enrollment, 95% confidence interval, the precision of 2.5% and 10% non-response rate, the estimated sample size was 1113.

A proportionate stratified sampling method was applied to ensure representativeness across all three states. According to data from the Department of Statistics Malaysia (DOSM), East Coast Malaysia had approximately 3.22 million people aged 18 and up [20]. The proportion of samples needed from Kelantan, Terengganu and Pahang were 427, 279, and 407 respectively. Samples at each stratum were then collected via convenience sampling.

### Research tool

The research tool is a bilingual proforma in English and Malay languages which were converted into Google Forms. The proforma contains questions on the state of residence, age, gender, ethnicity, marital status, educational level, employment status, household income category, location of residence, PHI subscription, and open-ended questions on the decision to purchase PHI. Ethnicity was divided into Malay and non-Malays due to low participation rates from other ethnicities. The educational level was divided into two categories: higher and lower education. Income cut-off points from the Household Income and Basic Amenities Survey Report were used to categorise households into three categories: bottom 40% (monthly household income less than MYR4850), middle 40% (monthly household income MYR4850-10971) and top 20% (monthly household income exceeding MYR10971) [21]. Locations of residence were classified based on population count; “rural” was defined as gazetted areas with fewer than 10,000 people, while “urban” was defined as areas with 10,000 or more people [22].

#### Ethical consideration

Ethical approval was obtained from the Human Research Ethics Committee of Universiti Sains Malaysia (USM/JEPeM/20110586) dated February 7^th^, 2021. The study information and written consent were provided before the initiation of the survey. Participants were informed that their participation in the study was entirely voluntary and that they could refuse or withdraw at any time without penalty or loss of benefit. By clicking "Yes" in the consent section of the study statement, participants were deemed to have agreed and consented to participate in the study. They were then directed to the pro forma questions. Those who did not want to participate could opt out by clicking "No" in the consent statement section and were not directed to the questions. Children under the age of 18 were not included in this study.

#### Data collection

The data was collected online, and the link to the survey was distributed through the research team members’ social network circles via platforms such as emails, WhatsApp, and Facebook pages. The link came with a brief explanation of the study’s objectives and selection criteria. Answering the pro forma questions took approximately five minutes. The study did not collect any personally identifiable information, and participants were kept anonymous. Accepting to participate was regarded as consenting to the study. The survey link remained active for two months, from February to March 2021.

#### Data analysis

The data analysis was performed using the Statistical Package for the Social Sciences (SPSS) version 26. The outcome was a binary variable indicating whether or not a person purchased a PHI policy. The independent variables were age, gender, educational level, marital status, ethnicity, household income, employment status, and residence.

Data were analyzed using descriptive statistics and chi-square tests. The result was presented in crude odds ratio (OR). Any independent variable with a p-value of less than 0.25 was selected for the multiple logistic regression analysis. Results were reported in adjusted OR (aOR) and 95% confidence interval. A p-value of less than 0.05 was considered significant. An OR greater than one indicated that the specified variable was more likely to be associated with the decision to purchase PHI.

## Results

### Characteristics of the participants

The study included a total of 1138 participants. Six hundred and eighteen participants (54.3%) had purchased PHI. The mean age of the study participants age was 36.88 years (SD 9.09). The majority of the participants were female (60.8%), Malay (96.4%), married (77.3%), had tertiary qualifications (85.6%), and worked in the public sector (70.9%). The difference in characteristics between PHI purchasers and non-purchasers was analysed using Chi-square tests of independence, and the results are shown in Table 1.

**Table 1:** Sociodemographic characteristics of the study participants (n = 1138).

Characteristics	No PHI		PHI		Total (n)
	n (%)		n (%)		
**Age**					
**18–24**	32	(84.2)	6	(15.8)	38
**25–34**	220	(41.1)	315	(58.9)	535
**35–44**	142	(42.9)	189	(57.1)	331
**45–54**	98	(53.8)	84	(46.2)	182
**55–64**	21	(50.0)	21	(50.0)	42
**> 65**	7	(70.0)	3	(30.0)	10
**State of residence**					
**Kelantan**	193	(44.6)	240	(55.4)	433
**Terengganu**	165	(54.8)	136	(45.2)	301
**Pahang**	162	(40.1)	242	(59.9)	404
**Gender**					
**Male**	211	(47.3)	235	(52.7)	446
**Female**	309	(44.7)	383	(55.3)	692
**Ethnicity**					
**Malay**	505	(46.0)	592	(54.0)	1097
**Non-Malay**	15	(36.6)	26	(63.4)	41
**Marital status**					
**Single**	123	(52.6)	111	(47.4)	234
**Married**	383	(43.5)	497	(56.5)	880
**Divorced/Widowed**	14	(58.3)	10	(41.7)	24
**Educational level**					
**Secondary or below**	106	(64.6)	58	(35.4)	164
**Tertiary**	414	(42.5)	560	(57.5)	974
**Employment status**					
**Public sector**	338	(41.9)	469	(58.1)	807
**Private sector**	87	(47.0)	98	(53.0)	185
**Self-employed**	34	(44.7)	42	(55.3)	76
**Not working**	61	(87.1)	9	(12.9)	70
**Household income percentile** ^a^					
**Bottom 40%**	241	(63.4)	139	(36.6)	380
**Middle 40%**	213	(40.1)	318	(59.9)	531
**Top 20%**	66	(29.1)	161	(70.9)	227
**Residence**					
**Urban**	263	(41.7)	368	(58.3)	631
**Rural**	257	(50.7)	250	(49.3)	507

^a^ The classification is based on the Household Income and Basic Amenities Survey Report [21]

#### Factors associated with the purchasing of private health insurance

The findings of the bivariable logistic regression analysis are shown in Table 2. Age, marital status, educational attainment, employment status, household income category, and location of residence were factors that the bivariable logistic model found to be associated with PHI uptake. The multivariable logistic regression analysis was performed by using enter (manual) methods. Three variables were identified as significant determinants for PHI uptake namely employment status, household income category, and location of residence. The two-way interaction between these three variables was checked and there was no significant interaction and no multicollinearity detected in the model. The variance inflation factor (VIF) was less than 10 and the tolerance was greater than 0.1.

**Table 2:** Logistic regression analysis of determinants of private health insurance uptake.

Variables	Crude OR (95% CI)		*p*-value^a^	aOR (95% CI)		*p*-value^b^
**Age (years)**						
**18–24**	1.00			1.00		
**25–34**	7.64	(3.14,18.57)	<0.001	2.67	(0.99,7.24)	0.053
**35–44**	7.10	(2.89,17.44)	<0.001	2.07	(0.74,5.80)	0.166
**45–54**	4.57	(1.82,11.46)	0.001	1.14	(0.40,3.29)	0.809
**55–64**	5.33	(1.85,15.41)	0.002	2.03	(0.61,6.71)	0.247
**> 65**	2.29	(0.46,11.43)	0.314	4.77	(0.70,32.65)	0.111
**Gender**						
**Male**	1.00					
**Female**	1.11	(0.88,1.41)	0.380			
**Ethnicity**						
**Malay**	1.00					
**Non-Malay**	1.48	(0.78,2.82)	0.236			
**Marital status**						
**Single**	1.00			1.00		
**Married**	1.44	(1.08,1.92)	0.014	0.86	(0.59,1.25)	0.424
**Divorced/Widowed**	0.79	(0.34,1.85)	0.590	0.69	(0.27,1.80)	0.446
**Educational level**						
**Secondary or below**	1.00			1.00		
**Tertiary**	2.47	(1.75,3.49)	<0.001	1.46	(0.98,2.17)	0.060
**Employment status**						
**Not working**	1.00			1.00		
**Public sector**	9.41	(4.61,9.20)	<0.001	6.06	(2.65,13.88)	<0.001
**Private sector**	7.64	(3.58,16.28)	<0.001	6.27	(2.65,14.85)	<0.001
**Self-employed**	8.37	(3.64,19.26)	<0.001	9.23	(3.59,23.70)	<0.001
**Income group**						
**Bottom 40%**	1.00			1.00		
**Middle 40%**	2.59	(1.97,3.40)	<0.001	2.74	(1.95,3.85)	<0.001
**Top 20%**	4.23	(2.97,6.03)	<0.001	4.42	(2.87,6.80)	<0.001
**Residence**						
**Rural**	1.00			1.00		
**Urban**	1.44	(1.14,1.82)	0.002	1.31	(1.01,1.70)	0.039

^a^ Bivariable Logistic Regression

^b^ Multivariable Logistic Regression

The Hosmer-Lemeshow goodness of fit test was not significant (p-value = 0.268), suggesting that the model was fit. The classification table indicated that the model correctly predicted 65.1% of the cases. The area under the Receiver Operating Characteristics (ROC) curve was 0.681 (95% CI: 0.650, 0.712), which means the model could accurately discriminate 68.1% of the cases (modest fit).

## Discussion

The total proportion of PHI uptake in the three states was 54.3%. Pahang had the highest proportion (21.3%), followed by Kelantan (21.1%), and Terengganu (12.0%). These findings were higher compared to those reported in the 2019 National Health and Morbidity Survey which reported the proportion of PHI uptake in Pahang, Kelantan, and Terengganu as 17.0%, 4.9%, and 9.7%, respectively [11]. It is postulated that the method of data collection may have contributed to the discrepancy between the two findings. The National Health and Morbidity Survey was a nationwide cross-sectional survey with a complex study design. Participants from all over the country were equally distributed in terms of socio-economic characteristics, which resulted in a much lower proportion. Whereas this study used convenience sampling and a web-based survey method which attracted younger, more active internet users, and financially stable and educated participants, thus contributing to a higher proportion.

This proportion was also considerably high when compared against China and Brazil, which reported lower proportions of PHI coverage of 5.0% and 13%, respectively [12, 13]. It is important to note, however, that the health financing mechanisms in both countries were dominated by mandatory health insurance schemes, which serve little need for voluntary PHI purchasing.

The proportion of PHI uptake was also influenced by the functional type of PHI. For instance, in France the PHI uptake was as high as 86% [23]. Because statutory health insurance only covers up to 75% of total health expenditures, complementary PHI is required to reimburse co-payments and medical products or services not covered by the public healthcare system [24]. In Malaysia, PHI is supplementary. Acquiring supplementary PHI guarantees expedited access to treatment, a greater selection of healthcare providers, and enhanced service or convenience, such as a private room [10, 25, 26].

In this study, the theoretical framework developed by Andersen was applied to categorise the multiple factors that influence PHI uptake, namely predisposing (age, gender, ethnicity, marital status, and level of education) and enabling factors (employment status, household income, and location of residence). Several authors have applied this model to comprehend the demand and purchase of PHI, despite the fact that the model was originally developed for the utilisation of health services [13, 27, 28]. According to the Andersen model, all three factors that were significantly associated with the participants’ decision to purchase PHI were enabling factors.

Firstly, employment status had a positive association with PHI uptake. As compared to those who were unemployed, participants who were currently working in both the public and private sectors had six times the odds of purchasing PHI, while those who were self-employed had nine times the odds of purchasing PHI. Such findings mirrored those in Brazil, China, Namibia, and South Korea [12–14, 18]. Understandably, individuals employed in certain sectors or holding important positions preferred seeking care at private hospitals to avoid the long waiting times at public hospitals, thus increasing the odds of purchasing the PHI. Having secure and stable employment was an important factor in deciding whether to acquire PHI, as it is associated with disposable personal income. Additionally, the types of occupations or employment also had some influence on the purchase of PHI. For example, a study in China found that employees of state-owned enterprises and private or foreign-owned enterprises were more likely to acquire PHI compared to other types of enterprises [13]. While in Brazil, the highest PHI uptake was seen among military personnel, followed by employees in the private and public sectors [12].

Secondly, household income was positively related to the likelihood of purchasing PHI as wealthy households have more disposable income to acquire PHI. Respectively, participants in the middle 40% and top 20% categories in this study had two and four times the odds of purchasing PHI compared to those in the bottom 40% category. The finding was consistent with studies in Namibia, Brazil, and China [12–14]. According to Odeyemi and Nixon (2013), high PHI coverage was associated with high socioeconomic status in developed countries such as Australia, the United States, the United Kingdom, Latvia, and France [29].

Thirdly, the current study discovered that urban residents had higher PHI coverage. The findings were similar to those in Namibia and China [12, 14]. The difference in PHI uptake between the urban-rural communities may be attributed to the distribution of private panel hospitals in Malaysia, which were more concentrated in the urban areas [30].

Age had no significant association with the decision to purchase PHI, though the distribution of PHI was concentrated among participants in the age group of 25–44 years. Consistent with a finding in China [13], it is believed that people in this age group are more financially stable compared to the younger generation (aged 18–24 years). Also, since PHI is risk-rated, PHI premiums for older age groups are more expensive, and deter older individuals from purchasing PHI.

Similar to a study conducted in Brazil [12], gender was not significantly associated with PHI uptake in the current study. However, female participants had a slightly higher uptake of PHI compared to their male counterparts (55.3% vs. 52.7%). This might be attributed to female empowerment, which led to greater involvement in decision-making, income-earning, and health concerns.

Even though findings in studies conducted in China and Kenya illustrated that being married increased the likelihood of acquiring PHI [13, 31], a similar outcome was not observed in the present study. It is hypothesised that with increasing awareness and improving health literacy, the status of PHI ownership will become independent of marital status.

Educational levels also had no association with PHI uptake among the east coast populations in Malaysia despite many studies showing contradicting findings [13, 16, 17]. However, participants who attained tertiary education had a higher percentage of PHI uptake compared to those with secondary education or below. Higher educational levels are generally associated with higher levels of health literacy People who are more educated have more opportunities to gain accurate information regarding their health status and the services provided in the community, thus improving their capability to evaluate the costs and benefits of acquiring PHI. In addition, people with higher education levels also tend to have better employment with promising salary prospects.

This study has several limitations. First, due to the generalisability of convenience sampling being unclear, estimates derived from convenience samples are often biased. As a result, while the findings of this study may not apply to the entire population of Malaysia, they may be indicative of the study participants. Second, internet surveys may be influenced by response bias, in which the truthfulness of responses cannot be guaranteed. Fortunately, the survey did not seek opinions and did not contain any scale items which prevented social desirability and extreme response bias from occurring.

## Conclusions

The high proportion of PHI uptake among the East Coast Malaysian population provides evidence that PHI is indeed in demand. As such, PHI may be considered a feasible option to reduce the burden of the public healthcare system. Despite their scepticism, Malaysians were still willing to purchase PHI. The Ministry of Health reiterated the role of PHI to complement and supplement the government in financing health services. More individuals who are self-employed, working in the public and private sectors, belonging to the middle- and high-income groups, and residing in urban areas should be encouraged to purchase PHI by increasing their awareness of the importance and benefits of PHI. Social media promotions and campaigns may be used to highlight how PHI can reduce the burden on public healthcare, lower out-of-pocket expenditure, and provide faster access and more options for healthcare treatment. Various measures have been instituted to assist the growth of the PHI industry, such as offering tax rebates for policyholders and introducing affordable PHI for government employees and the public. However, PHI is primarily profit-motivated and has no concern for the ultimate universal health coverage goals. Therefore, before this initiative can be adopted, it is crucial to ensure strict regulations and monitoring are in place to protect the rights of PHI policyholders.

## Supporting information

S1 Dataset (SAV)
